# Highly favorable physiological responses to concurrent resistance and high-intensity interval training during chemotherapy: the OptiTrain breast cancer trial

**DOI:** 10.1007/s10549-018-4663-8

**Published:** 2018-01-18

**Authors:** Sara Mijwel, Malin Backman, Kate A. Bolam, Emil Olofsson, Jessica Norrbom, Jonas Bergh, Carl Johan Sundberg, Yvonne Wengström, Helene Rundqvist

**Affiliations:** 1 Department of Physiology and Pharmacology, Karolinska Institutet, Stockholm, Sweden; 2 Department of Neurobiology, Care Sciences and Society, Karolinska Institutet, Stockholm, Sweden; 3 School of Human Movement and Nutrition Sciences, The University of Queensland, Brisbane, Australia; 4 Department of Cell and Molecular Biology, Karolinska Institutet, Stockholm, Sweden; 5 Department of Oncology and Pathology Cancer Center Karolinska, Karolinska Institutet, Stockholm, Sweden; 6 Cancer Theme, Karolinska University Hospital, Stockholm, Sweden; 7 Department of Learning, Informatics, Management and Ethics, Karolinska Institutet, Stockholm, Sweden; 8 Division of Nursing, Karolinska University Hospital, Stockholm, Sweden

**Keywords:** Breast cancer, High-intensity interval training, Chemotherapy, Pressure-pain threshold

## Abstract

**Background:**

Advanced therapeutic strategies are often accompanied by significant adverse effects, which warrant equally progressive countermeasures. Physical exercise has proven an effective intervention to improve physical function and reduce fatigue in patients undergoing chemotherapy. Effects of high-intensity interval training (HIIT) in this population are not well established although HIIT has proven effective in other clinical populations. The aim of the OptiTrain trial was to examine the effects of concurrent resistance and high-intensity interval training (RT-HIIT) or concurrent moderate-intensity aerobic and high-intensity interval training (AT-HIIT), to usual care (UC) on pain sensitivity and physiological outcomes in patients with breast cancer during chemotherapy.

**Methods:**

Two hundred and forty women were randomized to 16 weeks of RT-HIIT, AT-HIIT, or UC. Outcomes: cardiorespiratory fitness, muscle strength, body mass, hemoglobin levels, and pressure-pain threshold.

**Results:**

Pre- to post-intervention, RT-HIIT (ES = 0.41) and AT-HIIT (ES = 0.42) prevented the reduced cardiorespiratory fitness found with UC. Handgrip strength (surgery side: RT-HIIT vs. UC: ES = 0.41, RT-HIIT vs. AT-HIIT: ES = 0.28; non-surgery side: RT-HIIT vs. UC: ES = 0.35, RT-HIIT vs. AT-HIIT: ES = 0.22) and lower-limb muscle strength (RT-HIIT vs. UC: ES = 0.66, RT-HIIT vs. AT-HIIT: ES = 0.23) were significantly improved in the RT-HIIT. Increases in body mass were smaller in RT-HIIT (ES = − 0.16) and AT-HIIT (ES = − 0.16) versus UC. RT-HIIT reported higher pressure-pain thresholds than UC (trapezius: ES = 0.46, gluteus: ES = 0.53) and AT-HIIT (trapezius: ES = 0.30).

**Conclusion:**

Sixteen weeks of RT-HIIT significantly improved muscle strength and reduced pain sensitivity. Both exercise programs were well tolerated and were equally efficient in preventing increases in body mass and in preventing declines in cardiorespiratory fitness. These results highlight the importance of implementing a combination of resistance and high-intensity interval training during chemotherapy for women with breast cancer.

## Introduction

The five-year relative breast cancer survival rate is continuously improving and approaching 90% in many countries [1]. Effective adjuvant therapies have the potential to substantially reduce recurrence and mortality but presents with significant side effects and quality of life consequences. The most commonly reported symptom during and after treatment for breast cancer is cancer-related fatigue (CRF), a multifactorial distress which together with treatment-induced pain contributes to physiological dysfunction [2]. The marked deterioration of the individual’s physical capacity after a cancer diagnosis is due to direct effects of chemotherapy on physical function as well as general reductions of activity levels and is associated with decreased hemoglobin levels (Hb) [3], reduced cardiorespiratory fitness, and declines in muscle strength [4].

Exercise interventions for patients undergoing curative breast cancer therapy have been well tolerated and have shown positive effects on physical function and CRF [4]. Previous findings from the OptiTrain trial [5] showed favorable effects of exercise training on CRF and self-reported pain; however, whether muscle strength and/or cardiorespiratory fitness is associated with CRF and/or pain remains to be elucidated. Moreover, patients with breast cancer usually exhibit hypersensitivity to pressure pain compared with healthy control subjects [6]. Chronic aerobic exercise training in healthy individuals has shown positive effects on pain tolerance but not on pressure-pain threshold (PPT) [7]. However, the hypoalgesic effect of acute exercise is well established [8].

Despite the increasing use of taxane-based treatment in addition to anthracycline-based treatment, and that taxane-related toxicities differ from those of taxane-free therapies [9], few studies have evaluated whether the exercise response differs depending on the type of chemotherapy [10]. Evidence-based exercise regimens with identified benefits are highly warranted by health care professionals and patients. In healthy individuals and in several pathological conditions, high-intensity interval training (HIIT) provides significant, time-efficient improvements in cardiorespiratory fitness [11] and pilot studies present HIIT as a safe training strategy also in patients with breast cancer [12]. In addition, HIIT may induce beneficial neuromuscular adaptations [13] and anti-inflammatory effects [14], both proposed as mechanisms contributing to fatigue [15]. The physiological outcomes of HIIT and whether it best combines with aerobic or resistance exercise during chemotherapy are currently unknown. While aerobic exercise provides established QoL improvements, recent studies emphasize incorporation of resistance training to address loss of muscle strength and function [4]. This in-clinic, randomized controlled trial is the first to incorporate and compare high-intensity interval training combined with either conventional resistance (RT-HIIT) or aerobic (AT-HIIT) exercise to usual care (UC) on cardiorespiratory fitness, muscular strength, body mass, Hb, and pressure-pain threshold. In addition, the association between CRF and pain with physiological outcomes was assessed.

## Materials and methods

### Participants

Between March 2013 and July 2016, two hundred and forty patients at the Karolinska University Hospital (Stockholm, Sweden) were eligible for and accepted participation in the OptiTrain study (NCT02522260) [16]. The flow of participants through the trial has been reported elsewhere [5]. Inclusion criteria were Swedish-speaking women, 18–70 years old, breast cancer stage I–IIIa, scheduled to undergo chemotherapy consisting of anthracyclines, taxanes, or a combination of the two. Exclusion criteria were cardiac pathologies (assessed by routine electrocardiogram and a questionnaire), major psychiatric disorders, or other concurrent malignant diseases. Ethical approval was obtained from the Regional Ethical Review Board in Stockholm (Dnr 2012/1347-31/1, 2012/1347-31/2, 2013/632-32, 2014/408-32). All participants gave written informed consent prior to enrollment.

### Randomization and blinding

The participants were randomly allocated by the Clinical Studies Unit at Radiumhemmet, Karolinska University Hospital (Stockholm, Sweden) to either RT-HIIT, AT-HIIT, or UC at a 1:1:1 ratio using a computer-generated assignment program blinded to the research team. Participants, exercise-supervisors, and outcome-assessors were not masked to group allocation. The first measurement took place 1 week prior to participants’ second chemotherapy session and intervention groups (RT-HIIT and AT-HIIT) commenced the exercise training 3 days after the second chemotherapy session. Due to the limited time to perform ECG and baseline measurements before the first chemotherapy session, the intervention was initiated after the second chemotherapy session.

### Exercise training intervention

Exercise groups trained twice per week for 16 weeks at the Karolinska University Hospital rehabilitation center. All sessions were supervised by an exercise physiologist or oncology nurse to ensure safety, correct technique, progression, and encourage adherence to exercise protocols. The program was extended for participants with delays in chemotherapy (RT-HIIT, *n* = 8, range: 15–35 days; AT-HIIT, *n* = 5, range 13–32 days). The details of the training protocols have been published elsewhere [5, 16]. In brief, the RT-HIIT group completed both resistance and high-intensity interval exercise during each session. Participants performed 2–3 sets of 8–12 repetitions at an intensity of 80% of the patients’ estimated 1-repetition maximum. To ensure progressive overload, loads were adjusted throughout the training period when participants were able to perform more than 12 repetitions. The AT-HIIT group commenced with 20 min of moderate-intensity continuous aerobic exercise at a rating of perceived exertion (RPE) of 13–15 on the Borg scale [17]. Both RT-HIIT and AT-HIIT concluded with 3 × 3 min bouts of HIIT at an RPE of 16–18 interspersed with ~ 1 min of recovery.

### Outcomes

The primary outcome in the OptiTrain trial is cancer-related fatigue (CRF) [16], and findings from both the unidimensional European Organization for Research and Treatment for Cancer Quality of Life Questionnaire (EORTC-QLQ-C30), and multidimensional Piper Fatigue Scale have previously been published [5]. In the present study, we report findings on physiological outcomes including muscle strength, cardiorespiratory fitness, pressure-pain threshold, hemoglobin levels, and body mass. Moreover, assessments of associations between changes in physiological outcomes and changes in self-reported cancer-related fatigue as well as pain as assessed by the EORTC-QLQ-C30 were performed. (The EORTC-QLQ-C30 tool is sensitive to change in patients receiving chemotherapy [18]).

Handgrip strength was assessed by a hydraulic hand dynamometer (JAMAR, SAEHAN Corporation, Changwon, S. Korea), and lower-limb muscle strength by isometric mid-thigh pull (Baseline leg dynamometer, Fabrication Enterprises Inc., White Plains, NY, USA). Cardiorespiratory fitness, measured as predicted peak oxygen uptake (VO_2peak_), was assessed by the Åstrand-Rhyming submaximal cycle test [19], which has been validated for this population [20]. Hb was measured in venous blood (Clinical Studies Unit, Karolinska University Hospital, Stockholm, Sweden). Pressure-pain threshold (PPT) was measured bilaterally on the middle trapezius and gluteus muscles with an electronic algometer (Somedic Sales AB, Hörby, Sweden). No analgesics were taken 24 h prior to measurement. The applied pressure was at a rate of approximately 50 kPa/s by a 1 cm^2^ probe. The mean PPT from the bilateral measurements at each anatomical site was used for analysis. All outcomes were measured at baseline and at 16 weeks.

### Activity measures

At baseline, objective activity patterns were assessed by an accelerometer (model GT3X ActiGraph^®^ Corp, Pensacola, Florida, USA) and analyzed using validated wear-time specifications and cut-offs for adults [21]. Calculation of attendance and adherence to the exercise regimen has been described elsewhere [5].

### Statistics

With fatigue as the primary outcome measure in the OptiTrain trial, a sample size of 65 patients per group was required, based on an effect size of 0.53, using a two-sided ANOVA test with a power of 80% at 5% significance level. We aimed to recruit 80 participants into each group to account for an expected 20% attrition rate. Variables were visually checked for normality through QQ-plots and histograms and were found to be normally distributed. Baseline medical and demographic characteristics of each group were summarized using descriptive statistics. Exact *χ*^2^ tests were used for categorical variables. For between-group analyzes, we used analysis of covariance, adjusted for baseline values. Repeated-measures ANOVA was used to test for within-group differences. Standardized effect sizes (ES) were calculated and interpreted as described previously [5, 22]. An intention-to-treat approach was used, and missing data (9%) were imputed using the expectation maximization method after being determined to be “missing completely at random” [23]. The expectation–maximization algorithm is based on group change and the individual baseline score. For the outcome predicted VO_2peak_, participants receiving beta-adrenergic blocking agents (*n* = 2), or not reaching the target heart rate of 120–170 beats per min (*n* = 2), were excluded from the analysis. For all outcomes, a subgroup analysis was performed, and data were stratified into patients receiving taxanes (TAX) or not receiving taxanes (non-TAX). Post hoc comparisons were conducted with Bonferroni correction for three pairwise comparisons between RT-HIIT, AT-HIIT, and UC. Pearson’s coefficient of correlation (*r*) was used to evaluate the association between changes in physiological outcomes and changes in self-reported CRF and pain. All tests were two-tailed and a *p* value of < 0.05 was required for significance.

## Results

All groups were balanced at baseline (Table 1) [5], and no significant baseline differences were found between participants who dropped out versus participants who completed the study. Attendance and adherence to the training program have previously been reported [5]. There was no difference in attendance to exercise sessions for participants receiving TAX compared to those receiving non-TAX. No adverse events prompting medical attention occurred during exercise sessions.

**Table 1:** Participant characteristics at baseline

	RT-HIIT *n* = 74	AT-HIIT *n* = 72	UC *n* = 60
	Mean ± SD	Mean ± SD	Mean ± SD
Age (years)	52.7 ± 10.3	54.4 ± 10.3	52.6 ± 10.2
Body mass (kg)	68.7 ± 11.3	67.7 ± 13.0	69.1 ± 11.0
Height	165.7 ± 6.7	165.3 ± 6.6	166.4 ± 7.0
SED (% of daily wear time)	63.7 ± 7.7	65.6 ± 6.2	66.6 ± 7.2
MVPA (% of daily wear time)	9.6 ± 2.8	8.3 ± 2.8	8.5 ± 4.3
	*n* (%)	*n* (%)	*n* (%)
Married or partnered	60.6	59.7	69.5
University completed	67.6	64.7	66.0
Current smokers	4.3	5.9	5.2
Employed	74.6	86.8	79.7
Postmenopausal	51.4	63.9	61.7
Tumor profile			
Triple negative	14.9	11.0	16.7
HER2+, ER+/−	21.6	30.2	20.0
HER2−, ER+	62.2	58.9	61.6
HER2−, ER−	1.4	0.0	1.7
Anthracycline-based therapy	40.6	37.0	41.7
Taxane-based therapy	59.4	63.0	58.3

*SD* standard deviation, *RT-HIIT* resistance and high-intensity interval training, *AT-HIIT* moderate-intensity aerobic and high-intensity interval training, *UC* usual care, *MVPA* objectively measured moderate- to vigorous intensity physical activity, *SED* objectively measured sedentary behavior

### Cardiorespiratory fitness, muscle strength, body mass, and Hb

**Fig. 1:**
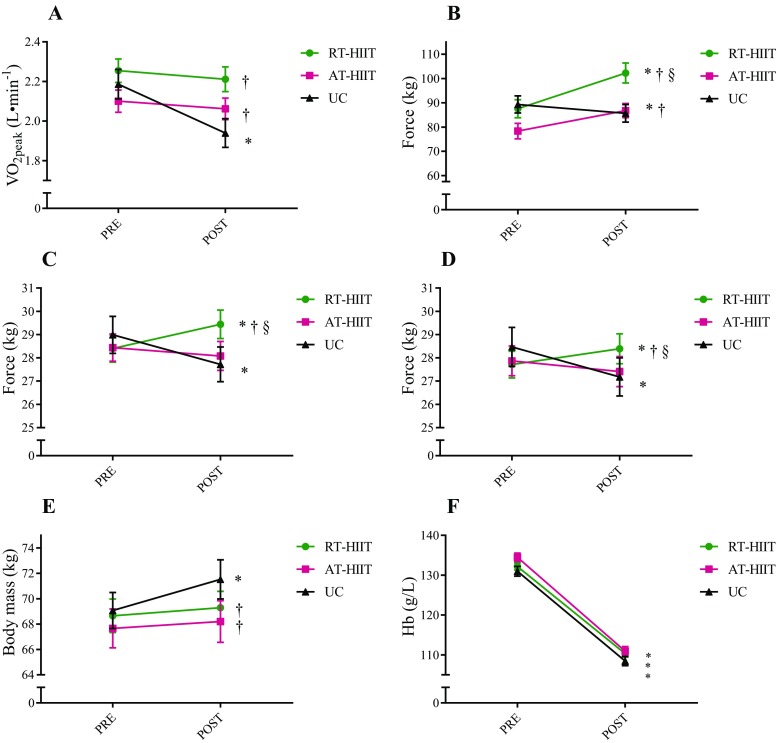
Effects of concurrent resistance and high-intensity interval training (RT-HIIT) and moderate-intensity aerobic and high-intensity interval training (AT-HIIT) versus usual care (UC) on physiological outcomes: **a** estimated VO_2peak_, **b** isometric mid-thigh pull, **c** handgrip strength surgery side, **d** handgrip strength non-surgery side, **e** body mass, and **f** hemoglobin levels. *p < 0.05 at post versus pre measurement; ^†^*p* < 0.05 compared to UC; ^§^*p* < 0.05 between RT-HIIT and AT-HIIT. Data is presented as mean and standard error of the mean. No statistically significant differences were found at baseline between groups

Changes in cardiorespiratory fitness, muscle strength, body mass, and Hb are shown in Fig. 1. Over the intervention, a significant decline in predicted VO_2peak_ was found for UC that was significantly different compared to unchanged levels for both RT-HIIT (ES = 0.41) and AT-HIIT (ES = 0.42). Participants in the UC group had significant handgrip strength losses, while significant handgrip muscle strength gains were found for the RT-HIIT group. Correspondingly, RT-HIIT was superior to both UC and AT-HIIT for handgrip strength (surgery side: RT-HIIT vs. UC: ES = 0.41, RT-HIIT vs. AT-HIIT: ES = 0.28; non-surgery side: RT-HIIT vs. UC: ES = 0.35, RT-HIIT vs. AT-HIIT: ES = 0.22). Both RT-HIIT and AT-HIIT significantly improved lower-limb muscle strength. The improved isometric mid-thigh pull strength test for RT-HIIT was significantly different from both UC (ES = 0.66) and AT-HIIT (ES = 0.23), and AT-HIIT was superior to UC (ES = 0.48). For estimated VO_2peak_ and muscle strength, similar effects were found for both patients receiving TAX and non-TAX (Table 2). The UC group had a significant weight gain from pre- to post-intervention that was different from the maintained body weight for both RT-HIIT and AT-HIIT (ES = − 0.16). Subgroup analyzes showed that for participants receiving TAX, only AT-HIIT maintained body mass, significantly different compared to increases in body mass in the UC group (ES = − 0.23), while for the non-TAX subgroup, only RT-HIIT prevented the increase in body mass found in the UC group (ES = − 0.17). Hb decreased similarly in all groups, regardless of treatment regimen, from the first to the last measurement.

**Table 2:** Mean values and standard deviations at the first measurement and at 16 weeks, and between-group differences for all outcome measures for participants receiving taxane- and non-taxane-based treatment

Outcome	Arm	*N*	Mean	SD	Mean	SD	*p* value
			Pre-intervention		Post-intervention		
Estimated VO_2peak_ Taxanes (L min^−1^)	RT-HIIT	43	2.24	0.53	2.23	0.60	0.845
	AT-HIIT	45	2.06	0.51	1.99	0.51	0.061
	UC	30	2.27	0.52	1.95	0.48	**<** **0.001**
Estimated VO_2peak_ Non-taxanes (L min^−1^)	RT-HIIT	30	2.27	0.48	2.18	0.43	0.140
	AT-HIIT	25	2.17	0.40	2.19	0.31	0.709
	UC	21	2.06	0.53	1.92	0.57	**0.001**
Isometric mid-thigh pull Taxanes (kg)	RT-HIIT	38	89.35	29.07	105.67	36.39	**<** **0.001**
	AT-HIIT	40	75.68	23.56	83.94	21.83	**<** **0.001**
	UC	30	90.07	20.08	85.47	21.92	0.153
Isometric mid-thigh pull Non-taxanes (kg)	RT-HIIT	27	84.26	30.52	96.27	26.91	**0.001**
	AT-HIIT	22	83.19	27.61	91.95	24.18	**0.003**
	UC	21	88.25	31.77	85.96	31.10	0.396
Handgrip surgery side Taxanes (kg)	RT-HIIT	43	28.16	4.98	29.49	5.33	**0.003**
	AT-HIIT	45	27.73	5.09	27.56	5.60	0.687
	UC	35	29.40	6.26	28.31	5.81	**0.018**
Handgrip non-surgery side Taxanes (kg)	RT-HIIT	43	27.54	4.69	28.47	5.33	**0.012**
	AT-HIIT	45	27.32	5.81	27.06	6.10	0.570
	UC	35	28.43	6.70	27.23	6.52	**0.011**
Handgrip surgery side Non-taxanes (kg)	RT-HIIT	31	28.73	5.18	29.35	5.26	0.169
	AT-HIIT	27	29.63	4.59	28.94	4.72	0.113
	UC	25	28.42	6.09	26.89	5.76	**0.033**
Handgrip non-surgery side Non-taxanes (kg)	RT-HIIT	31	27.95	5.32	28.27	5.90	0.540
	AT-HIIT	27	28.78	4.71	28.00	4.28	0.139
	UC	25	28.52	6.34	27.12	6.20	0.064
Body mass Taxanes (kg)	RT-HIIT	43	67.16	9.96	68.31	10.19	**0.008**
	AT-HIIT	45	67.40	10.97	67.61	10.93	0.514
	UC	35	68.77	10.02	71.46	10.46	**<** **0.001**
Body mass Non-taxanes (kg)	RT-HIIT	31	70.72	12.89	70.64	12.44	0.868
	AT-HIIT	27	68.10	16.05	69.18	18.06	0.073
	UC	25	69.48	12.38	71.62	13.94	**0.002**
Hemoglobin Taxanes (g/L)	RT-HIIT	43	133.42	7.04	108.68	7.33	**<** **0.001**
	AT-HIIT	45	135.00	7.96	108.85	9.61	**<** **0.001**
	UC	35	131.29	9.82	106.60	7.91	**<** **0.001**
Hemoglobin Non-taxanes (g/L)	RT-HIIT	31	130.42	7.88	112.55	7.36	**<** **0.001**
	AT-HIIT	27	133.59	10.59	114.39	9.68	**<** **0.001**
	UC	25	130.56	9.43	111.05	9.97	**<** **0.001**
PPT trapezius Taxanes (kPa)	RT-HIIT	41	424.67	159.92	441.65	163.41	0.374
	AT-HIIT	45	396.12	135.40	381.91	109.08	0.427
	UC	35	395.39	139.88	401.13	127.37	0.681
PPT gluteus Taxanes (kPa)	RT-HIIT	41	408.29	162.91	434.36	129.89	0.214
	AT-HIIT	45	410.69	195.77	400.39	128.21	0.631
	UC	35	434.66	153.58	415.69	132.43	0.349
PPT trapezius Non-taxanes (kPa)	RT-HIIT	29	411.43	115.63	457.95	115.63	0.053
	AT-HIIT	25	421.78	185.3	398.95	146.09	0.365
	UC	24	410.52	127.52	314.89	102.69	**<** **0.001**
PPT gluteus Non-taxanes (kPa)	RT-HIIT	29	437.86	114.38	450.39	142.29	0.503
	AT-HIIT	25	443.58	201.15	436.37	173.63	0.773
	UC	24	420.83	128.67	309.84	130.19	**<** **0.001**

Outcome	Adjusted mean change (95% CI):RT-HIIT versus UC	*p* value	ES	Adjusted mean change (95% CI):AT-HIIT versus UC	*p* value	ES	Adjusted mean change (95% CI):RT-HIIT versus AT-HIIT	*p* value	ES
Estimated VO_2peak_ Taxanes (L min^−1^)	0.30 (0.13 to 0.48)	**<** **0.001**	0.59	0.22 (0.04 to 0.39)	**0.013**	0.49	0.09 (− 0.07 to 0.25)	0.554	0.12
Estimated VO_2peak_ Non-taxanes (L min^−1^)	0.10 (− 0.08 to 0.29)	0.516	0.10	0.19 (0.01 to 0.38)	**0.047**	0.13	− 0.09 (− 0.26 to 0.09)	0.656	− 0.04
Isometric mid-thigh pull Taxanes (kg)	20.88 (11.92 to 29.85)	**<** **0.001**	0.82	12.08 (2.98 to 21.18)	**0.005**	0.58	8.80 (0.26 to 17.34)	**0.041**	0.30
Isometric mid-thigh pull Non-taxanes (kg)	13.52 (4.13 to 22.90)	**0.002**	0.46	10.05 (0.20 to 19.90)	**0.044**	0.37	3.47 (− 5.79 to 12.72)	1.000	0.11
Handgrip surgery side Taxanes (kg)	2.30 (0.80 to 3.80)	**0.001**	0.43	0.76 (− 0.73 to 2.24)	0.661	0.16	1.55 (0.15 to 2.94)	**0.025**	0.30
Handgrip non-surgery side Taxanes (kg)	2.08 (0.59 to 3.57)	**0.003**	0.37	0.87 (− 0.61 to 2.34)	0.471	0.15	1.21 (− 0.18 to 2.60)	0.109	0.22
Handgrip surgery side Non-taxanes (kg)	2.20 (0.48 to 3.91)	**0.007**	0.38	1.01 (− 0.77 to 2.79)	0.509	0.16	1.19 (− 0.50 to 2.87)	0.264	0.27
Handgrip non-surgery side Non-taxanes (kg)	1.63 (− 0.31 to 3.57)	0.128	0.30	0.66 (− 1.34 to 2.66)	1.000	0.11	0.97 (− 0.93 to 2.88)	0.642	0.22
Body mass Taxanes (kg)	− 1.57 (− 3.19 to 0.06)	0.062	− 0.15	− 2.50 (− 4.10 to − 0.89)	**0.001**	− 0.23	0.93 (− 0.59 to 2.45)	0.420	0.09
Body mass Non-taxanes (kg)	− 2.30 (− 4.15 to − 0.44)	**0.010**	− 0.17	− 0.99 (− 2.91 to 0.93)	0.628	− 0.07	− 1.30 (− 3.13 to 0.52)	0.254	− 0.08
Hemoglobin Taxanes (g/L)	1.07 (− 3.08 to 5.22)	1.000	0.01	0.49 (− 3.66 to 4.64)	1.000	0.17	0.58 (− 3.30 to 4.46)	1.000	0.19
Hemoglobin Non-taxanes (g/L)	1.56 (− 3.85 to 6.96)	1.000	0.19	2.14 (− 3.49 to 7.76)	1.000	0.03	− 0.58 (− 5.93 to 4.77)	1.000	− 0.14
PPT trapezius Taxanes (kPa)	21.7 (− 33.0 to 76.4)	1.000	0.07	− 19.7 (− 73.1 to 33.7)	1.000	− 0.13	41.4 (− 9.9 to 92.7)	0.158	0.19
PPT gluteus Taxanes (kPa)	31.6 (− 23.8 to 87.0)	0.507	0.27	− 3.6 (− 57.8 to 50.7)	1.000	− 0.06	35.2 (− 16.7 to 87.0)	0.308	0.18
PPT trapezius Non-taxanes (kPa)	142.5 (77.6 to 207.4)	**<** **0.001**	1.17	77.4 (10.2 to 144.7)	**0.019**	0.45	65.1 (0.9 to 129.3)	**0.046**	0.45
PPT gluteus Non-taxanes (kPa)	129.4 (57.9 to 198.8)	**<** **0.001**	1.02	110.3 (37.3 to 184.3)	**0.001**	0.61	18.1 (− 51.5 to 87.7)	1.000	0.12

*SD* standard deviation, *ES*, effect size, *PPT* pressure-pain threshold, *VO*_*2peak*_ peak oxygen consumption, *RT-HIIT* resistance and high-intensity interval training, *AT-HIIT* moderate-intensity aerobic and high-intensity interval training, *UC* usual care, *p* < 0.05 is highlighted in bold

### Pressure-pain threshold

**Fig. 2:**
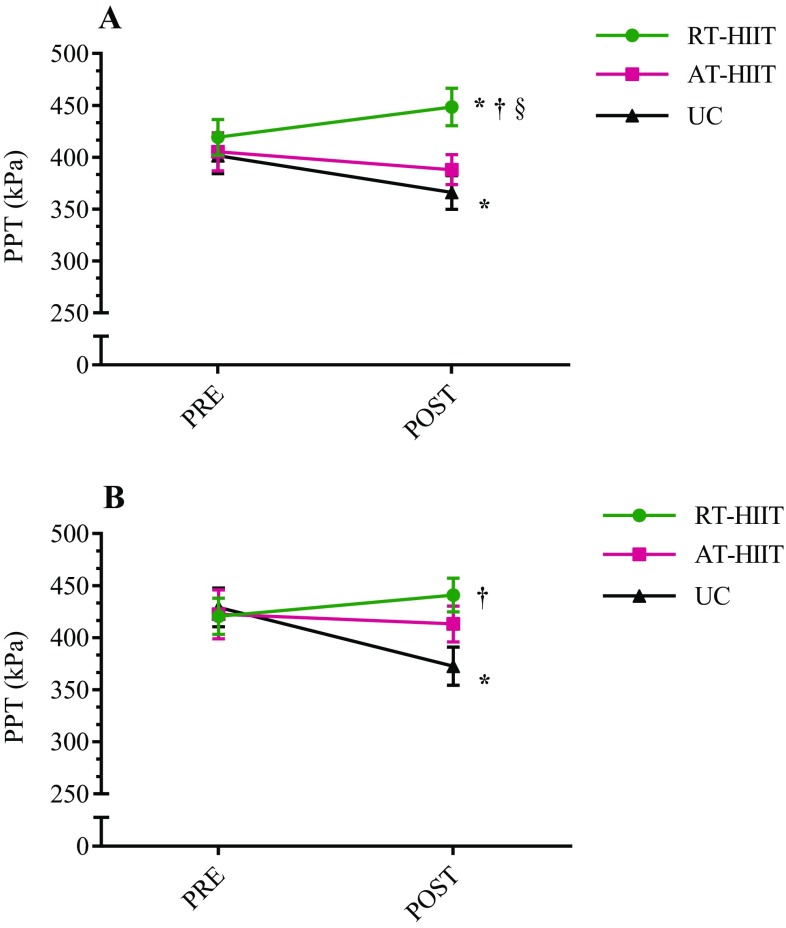
Pressure-pain thresholds (PPT) for **a** trapezius muscle, **b** gluteus muscle. *RT-HIIT* resistance and high-intensity interval training, *AT-HIIT* moderate-intensity aerobic and high-intensity interval training, *UC* usual care. *p < 0.05 at post versus pre measurement; ^†^*p* < 0.05 compared to UC; ^§^*p* < 0.05 between RT-HIIT and AT-HIIT. Data is presented as mean and standard error of the mean. No statistically significant differences were found at baseline between groups

Changes in PPT are shown in Fig. 2 and results from the subgroup analysis are shown in Table 2. Over the intervention, PPT measurements showed significant reductions for UC at both trapezius and gluteus muscles while RT-HIIT showed an increased PPT at the trapezius muscle, different when compared to UC (ES = 0.46) and AT-HIIT (ES = 0.30). PPT at the gluteus muscle favored RT-HIIT (ES = 0.53) compared to UC. For patients receiving non-TAX treatment, the UC group displayed significant reductions in PPT for both trapezius and gluteus muscles. For trapezius muscle, PPT for RT-HIIT was superior to both UC (ES = 1.17) and AT-HIIT (ES = 0.45), although AT-HIIT was still significantly different compared to UC (ES = 0.45). Significant differences were also found for PPT at the gluteus muscle favoring both RT-HIIT (ES = 1.02) and AT-HIIT (ES = 0.61) compared to UC. Patients receiving TAX treatment did not change in PPT and no between-group differences were found.

### Associations between physiological outcomes and self-reported cancer-related fatigue (CRF) and pain

Associations between changes in physiological outcomes and changes in self-reported cancer-related fatigue and pain are shown in Table 3. A weak inverse correlation was found between change in self-reported CRF and change in lower-limb strength (*r* = − 0.28, *p* < 0.001), and between change in self-reported CRF and change in PPT at the gluteus muscle (*r* = − 0.24, *p* = 0.001). No significant associations were found between change in self-reported fatigue and change in handgrip strength (surgery side *r* = − 0.12, *p* = 0.09; non-surgery side *r* = − 0.13, *p* = 0.06), or change in self-reported fatigue and change in cardiorespiratory fitness (*r* = − 0.10, *p* = 0.16). Similar associations were found for change in self-reported CRF measured by the Piper Fatigue Scale and change in physiological outcome measures (data not shown). Moreover, we found a significant correlation between changes in lower-limb muscle strength and changes in PPT at trapezius (*r* = 0.17, *p* = 0.03) and gluteus muscles (*r* = 0.16, *p* = 0.04), as well as between change in handgrip strength (non-surgery side) and change in PPT trapezius (*r* = 0.17, *p* = 0.02). Self-reported CRF was significantly associated with self-reported pain (*r* = 0.37, *p* < 0.001); however, no associations were found between physiological outcomes and self-reported pain.

**Table 3:** Pearson product-moment correlations between change in cancer-related fatigue and pain (EORTC-QLQ-C30) and change in physical outcome measures

Outcome	1	2	3	4	5	6	7	8
1. Self-reported cancer-related fatigue	–							
2. Self-reported pain	0.37*	–						
3. Cardiorespiratory fitness	− 0.10	0.00	–					
4. Lower-limb muscle strength	− 0.28*	− 0.07	0.25*	–				
5. Handgrip strength (surgery side)	− 0.12	− 0.07	0.15*	0.23*	–			
6. Handgrip strength (non-surgery side)	− 0.13	− 0.05	0.22*	0.22*	0.67*	–		
7. Pressure-pain threshold (trapezius muscle)	− 0.09	0.03	0.01	0.17*	0.10	0.17*	–	
8. Pressure-pain threshold (gluteus muscle)	− 0.24*	− 0.02	0.03	0.16*	− 0.02	0.11	0.64*	–

*EORTC-QLQ-C30* European Organization for Research and Cancer Treatment for Cancer Quality of Life Questionnaire, *p < 0.05

## Discussion

This is the first randomized controlled trial to include high-intensity interval training (HIIT) in two different exercise regimens in patients with breast cancer during chemotherapy. Results from this trial provide important updates in our understanding of potential management strategies for treatment-induced fatigue and physical dysfunction in this relatively large subgroup of cancer patients. This is also the first trial to investigate the ability of exercise training to prevent chemotherapy-induced hyperalgesia. Concurrent resistance and high-intensity interval training (RT-HIIT) provided significant beneficial effects in terms of counteracting cancer-related physical and total fatigue [5], reducing anthracycline-induced pain-hypersensitivity, and improving muscle strength in patients undergoing adjuvant treatment for breast cancer.

In concordance with previous findings, aerobic training resulted in sustained cardiorespiratory fitness [4]. Interestingly, cardiorespiratory fitness in the RT-HIIT group was maintained despite only a total of 9 min of HIIT per session, contradicting the results from Courneya et al. [24], where only the higher dose aerobic training (50–60 min, 3 days/week, 60–75% of VO_2peak_) resulted in a maintained cardiorespiratory fitness. This suggests that HIIT provides an effective and time-saving training strategy resulting in the same beneficial preservation of cardiorespiratory fitness as high-volume aerobic training, similar to findings in healthy individuals [11]. Results from the subgroup analyses showed that the declines in VO_2peak_ (L min^−1^) for the UC group were more severe for those receiving taxanes (–14%) compared to those not receiving taxanes (–7%). A previous exercise trial including periodic high and low-intensity components as well as relaxation exercise training reported on the challenges of preserving cardiorespiratory fitness during taxane-based treatment and found significant declines in cardiorespiratory fitness for both exercise and control groups [25]. In the current trial, both exercise interventions were able to counteract the decline in estimated VO_2peak_ in patients receiving taxanes, suggesting the importance of HIIT performed throughout the treatment period.

We previously showed that UC reported an increase in CRF measured by the EORTC-QLQ-C30 over the intervention, while no increase was found in the RT-HIIT and AT-HIIT groups [5]. Moreover, RT-HIIT was able to counteract the increase in physical and total CRF found in the UC group as measured by the Piper Fatigue Scale [5]. This is in line with previous trials showing that exercise training during chemotherapy was able to counteract CRF [4, 26]. Suggested mechanisms for CRF comprise reduced cardiorespiratory fitness and Hb [3]. Given the general decline in Hb and that aerobic fitness was equally maintained in both exercise groups, the physical CRF component is most likely multifactorial. It is currently unknown whether CRF is mainly induced by central or peripheral factors; however, recent studies point toward CRF being centrally mediated [27]. Studies on patients with advanced stage cancer suffering from CRF showed that these patients were unable to voluntarily recruit as much muscle as healthy controls [27], and had an earlier motor task failure [28]. The resistance-training component of RT-HIIT likely induced central neuromuscular adaptations through increased motor neuron firing frequencies [29]. In line with this, we found a weak inverse association between change in self-reported CRF and change in lower-limb muscle strength (*r* = − 0.28; *p* < 0.001). This is in concordance with findings from a study on patients with advanced cancer [30]. Weak negative associations have been found between maximal handgrip strength and self-reported CRF in breast cancer survivors [31]. We did not however find associations between handgrip strength and cardiorespiratory fitness measures with changes in self-reported CRF. A study by Thorsen et al. also failed to show associations between cardiorespiratory fitness (assessed by the same submaximal cycle test as in our trial) and self-reported CRF [32]. It may be speculated that stronger associations would be found between time to fatigue assessments and CRF. However, based on the current assessments, our findings indicate that lower-limb muscle strength may be an important underlying component of the multifactorial symptom CRF. Given that CRF can persist for up to ten years after a cancer diagnosis, and predicts shorter survival [3], implementing combined high-intensity intervals with resistance exercise regimens, similar to RT-HIIT, during chemotherapy may be critical for reducing the burden of this symptom.

In agreement with previous resistance-training interventions [4], we found a moderate effect size (0.66) for changes in lower-limb muscle strength with RT-HIIT compared to UC. Importantly, lower-limb muscle strength was also improved in the AT-HIIT group, not commonly reported with conventional aerobic exercise, supporting a role for HIIT in inducing neuromuscular adaptations. The handgrip strength improvements displayed by the RT-HIIT group offers a prognostic value and is an important correlate of health in survivors of breast cancer [31]. In the current study, strength gains were found with RT-HIIT regardless of chemotherapy regimen, and even slightly greater improvements for those receiving taxane-treatment for lower-limb muscle strength (+ 18.3%) compared to those on taxane-free treatment (+ 14.2%). Our findings are in contrast to those of Courneya [10], reporting that participants in a combined resistance and aerobic training group receiving taxane-treatment did not gain as much muscle strength as those not receiving taxanes. The discrepancy is unclear; however, despite that taxanes induce higher rates of myalgia, arthralgia, and neurosensory effects [9], a recent preclinical study showed no impairments in muscle function in response to taxanes [33].

Women with breast cancer commonly gain about 5 kg body weight during chemotherapy, and few return to their pre-diagnosis weight [34]. Here, no weight gain was observed in either of the intervention groups, except for the RT-HIIT group that received taxanes. The UC group gained body weight regardless of chemotherapy regimen. Since weight gain is associated with comorbidities and recurrence [34], maintaining pre-diagnosis weight is of major importance.

Physical fitness is associated with a high pressure-pain threshold (PPT) [35]. In the current study, the RT-HIIT intervention could completely compensate for the reduced PPT found in the UC group over the intervention. Notably, subgroup analyses showed that the patients receiving taxanes displayed no hyperalgesia over time, despite taxanes being more neurotoxic and associated with higher levels of self-reported pain compared to non-taxane treatments [36]. We speculate that taxanes may cause increased numbness leading to a blunted PPT response. In line with this, loss of vibratory perception has been reported with taxane therapies [37]. Moreover, we found no associations between self-reported pain and PPT. Our findings are in line with a previous study showing no associations between self-reported pain and heat pain threshold in healthy participants [38]. The same study noted that those who reported more pain were found to be more anxious than those who reported less pain, while objectively measured heat pain thresholds did not follow the same pattern. Given that a cancer diagnosis is accompanied by feelings of depression, fear, and anxiety [39], it may be speculated that the participants in our study were influenced by negative affect/anxiety that might have influenced their subjective feeling of pain, which could explain the lack of association between self-reported pain and PPT in the current study as well.

Of note, we found significant weak-to-moderate associations between changes in PPT at the gluteus and trapezius muscles and changes in lower-limb muscle strength as well as between changes in PPT at the trapezius muscle and changes in handgrip strength, indicating that muscle strength/function may be of particular importance in preserving objectively measured pain sensitivity. In contrast, a recent study assessing associations between PPT and muscle strength found no significant correlations [40].

The OptiTrain study is a sufficiently powered, supervised, in-clinic, randomized intervention trialing two types of progressive exercise regimens with validated measures. Our attendance rates are within the range commonly reported in exercise trials [4]. Limitations comprise that the first assessment was performed after one cycle of chemotherapy; however, this also provides some benefit by excluding acute effects of chemotherapy from analyses comparing first and last measurements. Moreover, a selection bias may have been introduced by the relatively large number of UC-patients declining participation directly after randomization. This aspect should generally be considered when drawing conclusions from exercise intervention studies.

## Conclusions

A 16-week supervised concurrent resistance and high-intensity interval training intervention (RT-HIIT) significantly improved muscle strength, and prevented hyperalgesia. Moreover, the RT-HIIT intervention was as efficient as AT-HIIT in maintaining body mass and cardiorespiratory fitness in women with early breast cancer receiving adjuvant chemotherapy. Findings from the current study also show that participants in the RT-HIIT group displayed similar beneficial effects from the exercise intervention regardless of receiving taxane or taxane-free treatment. This renders us to recommend that women receiving chemotherapy for primary breast cancer should be provided with knowledge of and access to a concurrent resistance and high-intensity interval training regimen.

## References

[1] International Agency for Research on Cancer: Cancer fact sheets Breast cancer (2016). https://www.iarc.fr/. Accessed 9 Mar 2017

[2] Davis MP, Walsh D (2010). Mechanisms of fatigue. J Support Oncol.

[3] Cramp F, Daniel J (2008). Exercise for the management of cancer-related fatigue in adults. Cochrane Database Syst Rev.

[4] Furmaniak AC, Menig M, Markes MH (2016). Exercise for women receiving adjuvant therapy for breast cancer. Cochrane Database Syst Rev.

[5] Mijwel S, Backman M, Bolam KA, Jervaeus A, Sundberg CJ, Margolin S, Browall M, Rundqvist H, Wengström Y (2017). Adding high-intensity interval training to conventional training modalities: optimizing health-related outcomes during chemotherapy for breast cancer: the OptiTrain randomized controlled trial. Breast Cancer Res Treat.

[6] Caro-Moran E, Fernandez-Lao C, Diaz-Rodriguez L, Cantarero-Villanueva I, Madeleine P, Arroyo-Morales M (2016). Pressure pain sensitivity maps of the neck–shoulder region in breast cancer survivors. Pain Med.

[7] Jones MD, Booth J, Taylor JL, Barry BK (2014). Aerobic training increases pain tolerance in healthy individuals. Med Sci Sports Exerc.

[8] Naugle KM, Fillingim RB, Riley JL (2012). A meta-analytic review of the hypoalgesic effects of exercise. J Pain.

[9] Ho MY, Mackey JR (2014). Presentation and management of docetaxel-related adverse effects in patients with breast cancer. Cancer Manag Res.

[10] Courneya KS, McKenzie DC, Mackey JR, Gelmon K, Reid RD, Friedenreich CM, Ladha AB, Proulx C, Vallance JK, Lane K, Yasui Y, Segal RJ (2008). Moderators of the effects of exercise training in breast cancer patients receiving chemotherapy: a randomized controlled trial. Cancer.

[11] Karlsen T, Aamot IL, Haykowsky M, Rognmo O (2017). High intensity interval training for maximizing health outcomes. Prog Cardiovasc Dis.

[12] Schmitt J, Lindner N, Reuss-Borst M, Holmberg HC, Sperlich B (2016). A 3 week multimodal intervention involving high-intensity interval training in female cancer survivors: a randomized controlled trial. Physiol Rep.

[13] Buchheit M, Laursen PB (2013). High-intensity interval training, solutions to the programming puzzle. Part II: anaerobic energy, neuromuscular load and practical applications. Sports Med.

[14] Steckling FM, Farinha JB, Santos DL, Bresciani G, Mortari JA, Stefanello ST, Courtes AA, Duarte T, Duarte MM, Moresco RN, Cardoso MS, Soares FA (2016). High Intensity Interval Training Reduces the Levels of Serum Inflammatory Cytokine on Women with Metabolic Syndrome. Exp Clin Endocrinol Diabetes.

[15] LaVoy ECP, Fagundes CP, Dantzer R (2016). Exercise, inflammation, and fatigue in cancer survivors. Exerc Immunol Rev.

[16] Wengström Y, Bolam KA, Mijwel S, Sundberg CJ, Backman M, Browall M, Norrbom J, Rundqvist H (2017). Optitrain: a randomised controlled exercise trial for women with breast cancer undergoing chemotherapy. BMC Cancer.

[17] Borg GA (1982). Psychophysical bases of perceived exertion. Med Sci Sports Exerc.

[18] Uwer L, Rotonda C, Guillemin F, Miny J, Kaminsky MC, Mercier M, Tournier-Rangeard L, Leonard I, Montcuquet P, Rauch P, Conroy T (2011). Responsiveness of EORTC QLQ-C30, QLQ-CR38 and FACT-C quality of life questionnaires in patients with colorectal cancer. Health Qual Life Outcomes.

[19] Åstrand I (1960). Aerobic work capacity in men and women with special reference to age. Acta Physiol Scand Suppl.

[20] Mijwel S, Cardinale D, Ekblom-Bak E, Sundberg CJ, Wengström Y, Rundqvist H (2016). Validation of 2 submaximal cardiorespiratory fitness tests in patients with breast cancer undergoing chemotherapy. Rehabil Oncol.

[21] Aguilar-Farias N, Brown WJ, Peeters GM (2014). ActiGraph GT3X+ cut-points for identifying sedentary behaviour in older adults in free-living environments. J Sci Med Sport.

[22] Morris SB (2007). Estimating effect sizes from pretest-posttest-control group designs. Organ Res Methods.

[23] Dong Y, Peng C-YJ (2013). Principled missing data methods for researchers. SpringerPlus.

[24] Courneya KS, McKenzie DC, Mackey JR, Gelmon K, Friedenreich CM, Yasui Y, Reid RD, Cook D, Jespersen D, Proulx C, Dolan LB, Forbes CC, Wooding E, Trinh L, Segal RJ (2013). Effects of exercise dose and type during breast cancer chemotherapy: multicenter randomized trial. JNCI J Natl Cancer Inst.

[25] Møller T, Lillelund C, Andersen C, Bloomquist K, Christensen KB, Ejlertsen B, Nørgaard L, Wiedenbein L, Oturai P, Breitenstein U, Adamsen L (2015). The challenge of preserving cardiorespiratory fitness in physically inactive patients with colon or breast cancer during adjuvant chemotherapy: a randomised feasibility study. BMJ Open Sport Exerc Med.

[26] van Waart H, Stuiver MM, van Harten WH, Geleijn E, Kieffer JM, Buffart LM, de Maaker-Berkhof M, Boven E, Schrama J, Geenen MM, Meerum Terwogt JM, van Bochove A, Lustig V, van den Heiligenberg SM, Smorenburg CH, Hellendoorn-van Vreeswijk JA, Sonke GS, Aaronson NK (2015). Effect of low-intensity physical activity and moderate- to high-intensity physical exercise during adjuvant chemotherapy on physical fitness, fatigue, and chemotherapy completion rates: results of the PACES Randomized Clinical Trial. J Clin Oncol.

[27] Yavuzsen T, Davis MP, Ranganathan VK, Walsh D, Siemionow V, Kirkova J, Khoshknabi D, Lagman R, LeGrand S, Yue GH (2009). Cancer-related fatigue: central or peripheral?. J Pain Symptom Manag.

[28] Kisiel-Sajewicz K, Davis MP, Siemionow V, Seyidova-Khoshknabi D, Wyant A, Walsh D, Hou J, Yue GH (2012). Lack of muscle contractile property changes at the time of perceived physical exhaustion suggests central mechanisms contributing to early motor task failure in patients with cancer-related fatigue. J Pain Symptom Manag.

[29] Aagaard P, Mayer F (2007). Neuronal adaptations to strength training. Deutsche zeitschrift für sportmedizin.

[30] Schvartsman G, Park M, Liu DD, Yennu S, Bruera E, Hui D (2017). Could objective tests be used to measure fatigue in patients with advanced cancer?. J Pain Symptom Manag.

[31] Cantarero-Villanueva I, Fernandez-Lao C, Diaz-Rodriguez L, Fernandez-de-Las-Penas C, Ruiz JR, Arroyo-Morales M (2012). The handgrip strength test as a measure of function in breast cancer survivors: relationship to cancer-related symptoms and physical and physiologic parameters. Am J Phys Med Rehabil.

[32] Thorsen L, Skovlund E, Strømme SB, Hornslien K, Dahl AA, Fosså SD (2005). Effectiveness of physical activity on cardiorespiratory fitness and health-related quality of life in young and middle-aged cancer patients shortly after chemotherapy. J Clin Oncol.

[33] Chaillou T, McPeek A, Lanner JT (2017). Docetaxel does not impair skeletal muscle force production in a murine model of cancer chemotherapy. Physiol Rep.

[34] Jiralerspong S, Goodwin PJ (2016). Obesity and breast cancer prognosis: evidence, challenges, and opportunities. J Clin Oncol.

[35] Lemming D, Borsbo B, Sjors A, Lind EB, Arendt-Nielsen L, Graven-Nielsen T, Gerdle B (2015). Single-point but not tonic cuff pressure pain sensitivity is associated with level of physical fitness–a study of non-athletic healthy subjects. PLoS ONE.

[36] Majithia N, Loprinzi CL, Smith TJ (2016). New practical approaches to chemotherapy-induced neuropathic pain: prevention, assessment, and treatment. Oncology.

[37] Hilkens PH, Verweij J, Vecht CJ, Stoter G, van den Bent MJ (1997). Clinical characteristics of severe peripheral neuropathy induced by docetaxel (Taxotere). Ann Oncol.

[38] Edwards RR, Fillingim RB (2007). Self-reported pain sensitivity: Lack of correlation with pain threshold and tolerance. Eur J Pain.

[39] Baqutayan SMS (2012). The effect of anxiety on breast cancer patients. Indian J Psychol Med.

[40] Alfieri FM, Lima ARS, Oliveira NC, Portes LA (2017). The influence of physical fitness on pressure pain threshold of elderly women. J Bodyw Mov Ther.

